# New medicines for spontaneous preterm birth prevention and preterm labour management: landscape analysis of the medicine development pipeline

**DOI:** 10.1186/s12884-023-05842-9

**Published:** 2023-07-18

**Authors:** Annie R. A. McDougall, Roxanne Hastie, Maya Goldstein, Andrew Tuttle, Anne Ammerdorffer, A. Metin Gülmezoglu, Joshua P. Vogel

**Affiliations:** 1 Maternal, Child and Adolescent Health Program, Burnet Institute, 85 Commercial Road, Melbourne, VIC 3004 Australia; 2 Department of Obstetrics and Gynaecology, University of Melbourne, Heidelberg, Australia; 3 Policy Cures Research, Sydney, Australia; 4 Concept Foundation, Geneva, Switzerland; 5 School of Public Health and Preventive Medicine, Monash University, Melbourne, Australia

**Keywords:** Aspirin, Celecoxib, Drug development, Isosorbide dinitrate, L-arginine, Nicardipine, Nicorandil, Omega-3 fatty acid, Oral progesterone, Preterm labour, Selenium, Tocolytics, Vaginal progesterone

## Abstract

**Background:**

There are few medicines in clinical use for managing preterm labor or preventing spontaneous preterm birth from occurring. We previously developed two target product profiles (TPPs) for medicines to prevent spontaneous preterm birth and manage preterm labor. The objectives of this study were to 1) analyse the research and development pipeline of medicines for preterm birth and 2) compare these medicines to target product profiles for spontaneous preterm birth to identify the most promising candidates.

**Methods:**

Adis Insight, Pharmaprojects, WHO international clinical trials registry platform (ICTRP), PubMed and grant databases were searched to identify candidate medicines (including drugs, dietary supplements and biologics) and populate the Accelerating Innovations for Mothers (AIM) database. This database was screened for all candidates that have been investigated for preterm birth. Candidates in clinical development were ranked against criteria from TPPs, and classified as high, medium or low potential. Preclinical candidates were categorised by product type, archetype and medicine subclass.

**Results:**

The AIM database identified 178 candidates. Of the 71 candidates in clinical development, ten were deemed high potential (*Prevention*: Omega-3 fatty acid, aspirin, vaginal progesterone, oral progesterone, L-arginine, and selenium; *Treatment*: nicorandil, isosorbide dinitrate, nicardipine and celecoxib) and seven were medium potential (*Prevention*: pravastatin and lactoferrin; *Treatment*: glyceryl trinitrate, retosiban, relcovaptan, human chorionic gonadotropin and *Bryophyllum pinnatum* extract). 107 candidates were in preclinical development.

**Conclusions:**

This analysis provides a drug-agnostic approach to assessing the potential of candidate medicines for spontaneous preterm birth. Research should be prioritised for high-potential candidates that are most likely to meet the real world needs of women, babies, and health care professionals.

**Supplementary Information:**

The online version contains supplementary material available at 10.1186/s12884-023-05842-9.

## Background

Preterm birth (born alive prior to 37 completed weeks’ gestation) is the leading cause of death in children under five and a major cause of newborn morbidity [1–3]. Up to 50% of preterm births are due to spontaneous preterm labour [4]. There are limited medicines in clinical use for spontaneous preterm birth/labour. Several drugs can delay preterm labour—a 2022 Cochrane network meta-analysis on tocolytics found all subclasses (betamimetics, COX inhibitors, calcium channel blockers, magnesium sulfate, oxytocin receptor antagonists, nitric oxide donors) are probably effective at delaying preterm birth by 48 h, and that calcium channel blockers possibly reduce the risk of some adverse neonatal outcomes including respiratory and neurodevelopmental morbidity [5]. The World Health Organization (WHO) subsequently recommended the calcium channel blocker nifedipine as the preferred tocolytic agent, however noted a lack of long-term follow up studies [6]. All tocolytic drugs in clinical use, apart from atosiban, are repurposed medicines that are used off-label in pregnant women [7]. Fewer options are available for preventing spontaneous preterm birth – while vaginal progesterone can prevent preterm birth in high- risk women (women a history of spontaneous preterm birth or shortened cervix), some clinical indications for use (short cervical length, detected via ultrasound) are not easy to identify in all settings [8]. In October 2022, the FDA recommended Makena (injectable 17-alpha-hydroxyprogesterone caproate) be withdrawn from market due to evidence of a lack of clinical benefit in preventing preterm birth [9, 10].

The lack of innovation in medicines for spontaneous preterm birth/labour can be attributed to the broader, long-standing lack of investment in research and development (R&D) for new medicines for obstetric conditions [11]. A 2014 analysis of maternal research funding demonstrated that very few funders, particularly in the pharmaceutical industry, place a high priority on maternal health medicines R&D [12]. In 2008 there were fewer drugs in active development for all maternal conditions than for the rare disease amyotrophic lateral sclerosis [13]. This “drug drought” in maternal medicines has meant only two new drugs—the tocolytic atosiban and carbetocin for prevention of postpartum haemorrhage – have been licenced over the past 30 years specifically for use among pregnant women [14].

The Accelerating Innovation for Mothers (AIM) project was initiated to catalyse the development of new medicines for obstetric conditions [15]. Within AIM, we previously developed two target product profiles (TPPs) for new medicines to prevent spontaneous preterm birth and manage preterm labour [16]. TPPs are strategic documents that describe the key characteristics of new products and have helped drive R&D in vaccines, diagnostics and therapeutics for multiple conditions [17–21]. In this study, we aimed to analyse a database established by the AIM project [22–24] of the pipeline of medicines for preterm birth and compared candidates to the TPPs to identify the most promising options for reducing preterm birth-related morbidity and mortality globally.

## Materials and methods

### AIM database of drug development pipeline

Development of the AIM medicine pipeline database has been previously described [22–25]. Briefly, the database was created by systematically searching leading pharmaceutical databases (Adis Insight and Pharmaprojects), WHO international clinical trials registry platform (ICTRP) and other clinical trial registries, PubMed and grant databases of top maternal health product funders to identify candidate medicines (including drugs, dietary supplements and biologics) investigated for five priority maternal conditions (preeclampsia, preterm birth/labour, post-partum hemorrhage, fetal growth restriction and fetal distress). The AIM project database identified a total of 444 candidates that were investigated during the period 2000 to 2021. Candidates under investigation for preterm birth/labour were included in the database regardless of aetiology of preterm birth. Preterm labor/birth had the largest number of candidates for any of the five pregnancy-related conditions, with 178 unique candidates.

To identify high-potential candidates in the pipeline, we applied a systematic, stepwise approach to assessing all 178 candidates for preterm birth prevention and preterm labour management. First, we excluded candidates that were: 1) approved and already available on the market for this indication; 2) recommended by WHO, or otherwise in routine clinical use for this indication; 3) already recommended or widely used to treat a subgroup of women within the condition of interest (for example, levothyroxine in pregnant women with hypothyroidism); 4) inactive due to negative trial outcomes (such as adverse maternal or neonatal outcomes); 5) indicated as inferior to current treatments based on currently available evidence; and 6) under investigation for other conditions related to labour/birth, such as labour induction, and not preterm birth.

### TPP matching of candidates in development phase I, II or III

We previously developed TPPs to guide development of novel medicines for prevention of spontaneous preterm birth and management of preterm labour – the first TPPs developed for preterm birth [26]. These TPPs included 21 parameters with “minimum” and “preferred” criteria defined for each parameter (Tables S1 and S2)—an ideal medicine would be one that met the preferred criteria for all 21 parameters. For the current analysis, we used a drug agnostic systematic matching approach [23], which utilises nine critical parameters from the TPPs as criteria to rank candidates in Phases I, II or III (Table 1, Table S1). These nine variables were selected based on their relative importance for wide-scale implementation, and the selection was informed by expert interviews during TPP development. TPP matching was performed for each candidate by two authors independently, and where differences arose, a third author was consulted to determine final matching.

**Table 1:** Critical parameters from target product profiles used to rank candidates in clinical development

**1. Setting** – Has the medicine been trialled for this indication in high-income country settings only, low-middle income country settings only, or both?
**2. Efficacy**—In the available trials for this indication, has the medicine demonstrated clinically significant effect on the efficacy outcome/s?
**3. Need for a companion diagnostic test**—In the available trials for this indication, has the medicine required the routine use of a companion diagnostic test?
**4. Need for clinical monitoring**—In the available trials for this indication, has the medicine required the use of routine monitoring, or additional clinical monitoring?
**5. Safety**—In the available trials for this indication, has the medicine demonstrated any safety concerns?
**6. Mode of administration** – In the available trials for this indication, what is the mode of administration? If no trials have been completed, what is the mode of administration for repurposed medicine?
**7. Treatment adherence**—In the available trials for this indication, what has been the adherence to treatment?
**8. Stability**—is cold chain required for this product?
**9. WHO Essential Medicines List**—Is the product is currently listed on the WHO Essential Medicines List or not?

Preclinical candidates were assessed descriptively, including categorisation by product type, new or repurposed, and medicine subclass. Comparison of preclinical candidates to the TPPs was not performed, given the lack of data for most of the TPP-based criteria.

### Data visualisation and ranking of potential

For each variable, candidates were assigned a numerical score representing the level of matching for a given variable of the TPP (Table 1 and Tables S1 and S2), as described in our previous study [23]. Given the greater importance of clinical efficacy and safety criteria, these variables were given a greater weight. These scores were also represented graphically – candidates were classified as having met preferred (dark green), met minimum (light green), partially met minimum (yellow) or did not meet minimum (red). Hence, the ranking of a candidate as high, medium or low is based on a systematic assessment of available evidence against pre-specified criteria.

## Results

Of the 178 candidates in the pipeline, seven (3.9%) were approved and on the market for the prevention or management of preterm birth (allylestrenol, atosiban, injectable 17-alpha-hydroxyprogesterone caproate, hexoprenaline, isoxsuprine, fenoterol, ritodrine). An additional 11/178 (6.2%) candidates are approved for other clinical conditions and have been used off-label for preterm birth (terbutaline, salbutamol, vaginal/topical progesterone, magnesium sulphate, orciprenaline, nicardipine, nifedipine, indomethacin, sulindac) or to promote fetal well-being following preterm delivery (dexamethasone, betamethasone).

**Fig. 1:**
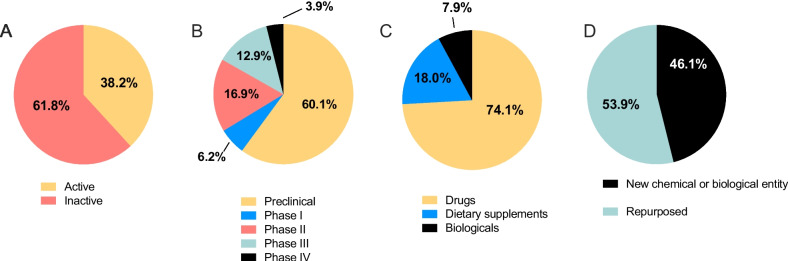
Details of the candidates in the R&D pipeline for preterm birth/labor. Summary of the 178 candidates in the R&D pipeline for the prevention of preterm birth and management of preterm labor from 2000 – 2021. The proportion of candidates **A** in active development, and inactive (no publications since 2018), **B** in each phase of the development pipeline, **C** classified as drugs, biologicals or dietary supplements, and **D** classified as new chemical or biological entities or repurposed drugs

**Fig. 2:**
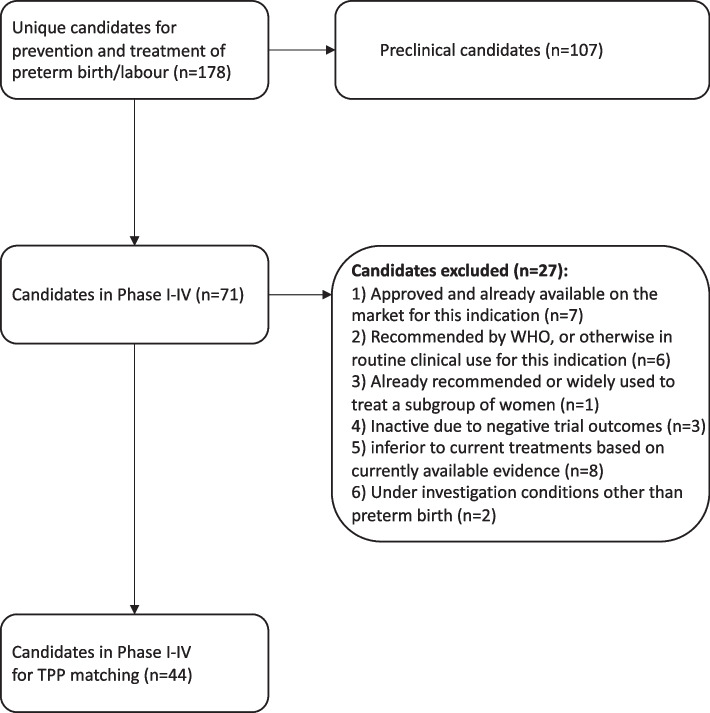
Flowchart of assessment of candidates against the eligibility criteria

In total, 68 (38.2%) candidates were currently active and 110 (61.8%) were inactive (no updates since 2018; Fig. 1A). The majority (107 candidates; 60.1%) were at the preclinical stage of development (Fig. 1B). In total, 11 candidates were in Phase I (6.2%), 30 candidates in Phase II (16.9%), 23 candidates in Phase III (12.9%) and 7 candidates in Phase IV (3.9%). Across all 178 candidates, 132 (74.1%) were classified as drugs, 14 (7.9%) were biologics and 32 (18.0%) were dietary supplements (Fig. 1C). In total, 82 (46.1%) were new chemical/biological entities and 96 (53.9%) were repurposed (Fig. 1D). Of the 71 candidates in clinical development, 27 were removed from further analysis due to the exclusion criteria, leaving 44 candidates (Fig. 2). In total, 10 clinical phase candidates were ranked as high potential, 7 as medium potential and 27 as low potential.

### Prevention of spontaneous preterm birth

**Fig. 3:**
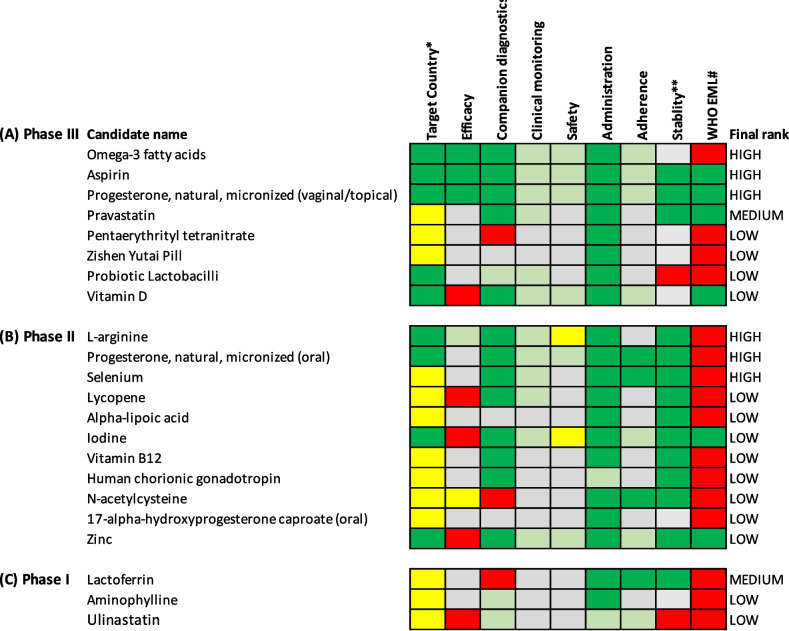
Visual representation of target product profile matching for candidates to prevent spontaneous preterm birth. A traffic light system to visualise each candidate for preterm birth prevention at **A** Phase III, **B** Phase II and **C** Phase I clinical development. Candidates are classified as met preferred (dark green), met minimum (light green), partially met minimum (yellow) and did not meet the minimum (red) requirements in the target product profiles. When insufficient information is available for a specific variable, they have been classified as not yet known (grey). *Target country is classified as trials being conducted in HIC and LMIC (dark green), HIC only or LMIC only (both yellow) or country not stated (grey). **Stability has been classified as does not require cold chain (green), requires cold chain (red) or unsure (grey). #WHO EML is classified as candidate is already on the WHO EML list (green), or candidate is not on the WHO EML list (red). Final rank has been determined by quantification of the matching to the target product profiles (see Tables S1 and S2 for details of quantification coding), with efficacy and safety given a greater weight than other variables. HIC = high-income country, LMIC = low- or middle-income country, EML = essential medicines list

Eight candidates were assessed for prevention in Phase III clinical trials (Fig. 3a), 10 in Phase II (Fig. 3b) and three in Phase I (Fig. 3c). Six candidates were ranked as high potential and two as medium potential. The evidence for candidates ranked low potential is presented in Additional file 2: Appendix B.

#### Phase III candidates

Aspirin, vaginal/topical progesterone and omega-3 fatty acids supplementation were ranked as high potential (Fig. 3a). A 2021 individual participant data meta-analysis found that vaginal progesterone reduces the risk of preterm birth (nine trials, 3769 women, RR 0.78, 95% CI 0.68 – 0.90) in women at increased risk, defined as women with a history of preterm birth and/or short cervix (< 25 mm) [8]. A 2018 Cochrane review examining omega-3 fatty acid supplementation during pregnancy found high quality evidence of a reduced risk of preterm birth < 37 weeks (26 trials, 10,304 women, RR 0.89, 95% CI 0.81 – 0.97) and < 34 weeks (9 trials, 5204 women, RR 0.58, 95% CI 0.44 – 0.77) [27]. Meta-analysis of 35 placebo-controlled trials found taking low dose aspirin during pregnancy reduced the risk of preterm birth (46,568 women, RR 0.90, 95% CI 0.86 – 0.94) [28], however, it is unclear if aspirin is beneficial in preventing spontaneous preterm birth, or if this benefit relates to the known effects of aspirin in reducing the risk of preeclampsia [29]. A 2017 individual participant data meta-analysis including 17 trials (including 28,797 women) evaluating aspirin for the prevention of preeclampsia, found that aspirin reduced the risk of spontaneous preterm birth at < 37 weeks (RR 0.93, 95% CI 0.86 – 0.996) and < 34 weeks (RR 0.86, 95% CI 0.76 – 0.99) [30]. Similar reductions were observed in a 2018 secondary analysis of a trial investigating the effect of low-dose aspirin to prevent preeclampsia. In women with a low risk of preeclampsia, aspirin reduced the odds of spontaneous preterm birth < 34 weeks’ gestation compared to placebo (OR 0.43, 95% CI 0.26 – 0.84; 2543 women) [31]. However, the 2022 APRIL trial assessed the effects of aspirin prophylaxis in 406 women with a history of preterm birth, and found no significant difference in the risk of preterm birth (RR 0.83, 95% CI 0.58 – 1.20) [32].

Pravastatin was ranked as medium potential (Fig. 3a). The clinical efficacy of pravastatin for preventing spontaneous preterm birth remains unknown. A 2021 meta-analysis of trials and observational studies found that any statin use during pregnancy was associated with a non-significant reduction in the risk of preterm birth (four studies, 483 women, OR 0.47, 95% CI 0.06 – 3.70), but also a significant increase in risk of spontaneous abortion (six studies, 4,165 women, OR 1.36, 95% CI 1.10 – 1.68) [33]. One mechanism by which pravastatin is thought to prevent preterm birth is by preventing preeclampsia [34], however a 2021 Phase III trial in 1120 women reported that pravastatin was not effective for this indication [35].

#### Phase II candidates

Oral progesterone, the amino-acid L-arginine and the trace element selenium were ranked high potential (Fig. 3b). A 2021 individual participant data meta-analysis of progesterone for preventing preterm birth found oral progesterone reduced the risk of preterm birth < 24 weeks (RR 0.60, 95% CI 0.41 – 0.90) [8]. However, this only included 2 trials of 183 women and more evidence is required to evaluate clinical efficacy. Meta-analysis of L-arginine supplementation in women at high risk of preeclampsia or with mild chronic hypertension showed it reduces the risk of preterm birth (three trials, 625 women, RR 0.50, 95% CI 0.30 – 0.85) [36]. While there are currently no clinical trials examining the effects of L-arginine supplementation on prevention of spontaneous preterm birth, a prospective cohort study of 7591 pregnant women in Tanzania found that the level of L-arginine dietary intake was associated with a decreased risk of spontaneous preterm birth [37].

Evidence of clinical efficacy of selenium is limited to a small trial in 180 HIV-positive pregnant women that found selenium reduced the risk of preterm birth (RR 0.32, 95% CI 0.11 – 0.96) [38]. Meta-analysis of observational data has also reported an association between prenatal maternal selenium plasma concentrations and reduced odds of preterm birth (17 cohorts, 9946 singleton births, OR 0.95, 95% CI 0.90 – 1.0) [39].

#### Phase I candidates

Lactoferrin was ranked as medium potential (Fig. 3c), and there is evidence from a small trial of 125 pregnant women with bacterial vaginosis that vaginal lactoferrin significantly reduced the rate of preterm birth (25.0% vs 44.6%, *p* = 0.02) [40]. However, lactoferrin did not meet the minimum requirements for companion diagnostics, as it requires a diagnosis of bacterial vaginosis, which involves laboratory testing of vaginal discharge to identify the causative agent. Additional laboratory diagnostic testing may act as a barrier to implementation in some settings.

### Management of preterm labour

**Fig. 4:**
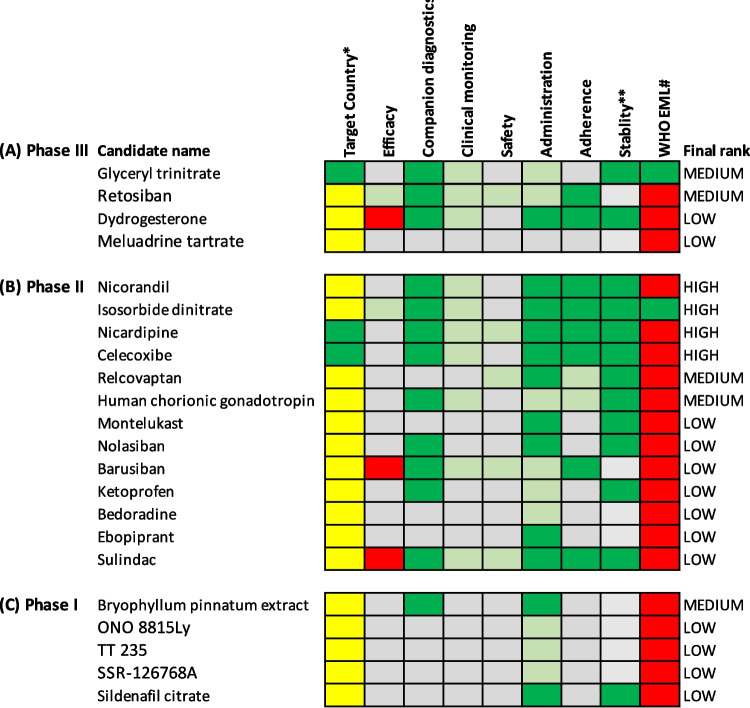
Visual representation of target product profile matching for candidates to manage preterm labor. A traffic light system to visualise each candidate for preterm labor treatment at **A** Phase III, **B** Phase II and **C** Phase I clinical development. Candidates are classified as met preferred (dark green), met minimum (light green), partially met minimum (yellow) and did not meet the minimum (red) requirements in the target product profiles. When insufficient information is available for a specific variable, they have been classified as not yet known (grey). *Target country is classified as trials being conducted in HIC and LMIC (dark green), HIC only or LMIC only (both yellow) or country not stated (grey). **Stability has been classified as does not require cold chain (green), requires cold chain (red) or unsure (grey). #WHO EML is classified as candidate is already on the WHO EML list (green), or candidate is not on the WHO EML list (red). Final rank has been determined by quantification of the matching to the target product profiles (see Tables S1 and S2 for details of quantification coding), with efficacy and safety given a greater weight than other variables. HIC = high-income country, LMIC = low- or middle-income country, EML = essential medicines list

There were four candidates assessed for management of preterm labour in Phase III (Fig. 4a), 13 in Phase II (Fig. 4b) and five in Phase I (Fig. 4c). Four candidates were ranked as high potential and five as medium potential. The evidence for candidates ranked low potential is presented in Additional file 2: Appendix B.

#### Phase III candidates

No Phase III candidates were identified as high potential. Retosiban, a selective oxytocin antagonist and glyceryl trinitrate, a nitric oxide donor were ranked medium potential (Fig. 4a). A pilot trial of 29 pregnant women suggested retosiban has a favourable safety and tolerability profile [41]. A small placebo-controlled trial of 64 women in preterm labour found retosiban significantly prolonged pregnancy by an average of 8.2 days and reduced the risk of preterm birth (RR 0.38, 95% CI 0.15 – 0.81) [42]. A Phase III trial of retosiban compared to placebo or atosiban was stopped early due to failure to recruit [43]. Data from this stopped trial suggested a possible increase in time to delivery compared to placebo (23 women) and no significant difference in time to delivery between retosiban and atosiban (97 women). The 2022 Cochrane network meta-analysis on tocolytics found that nitric oxide donors such as Glyceryl trinitrate (13 trials) probably delay preterm birth by 48 h (RR 1.17, 95% CI 1.05 – 1.31) and 7 days (RR 1.18, 95% CI 1.02 – 1.37) [5].

#### Phase II candidates

Nicardipine, nicorandil, isosorbide dinitrate and celecoxib were all ranked high potential and are all from the same drug class as tocolytics in clinical use (Fig. 4b). Nicardipine and nicorandil are calcium channel blockers, isosorbide dinitrate is a nitric oxide donor and celecoxib is a COX-2 inhibitor. The 2022 Cochrane network meta-analysis found that calcium channel blockers, nitric oxide donors and COX-2 inhibitors were all effective in delaying preterm birth compared to placebo or no tocolytic [5]. COX inhibitors and calcium channel blockers are both possibly effective at delaying birth by 48 h (RR 1.11, 95% CI 1.01 – 1.23, and RR 1.16, 95% CI 1.07 – 1.24, respectively). Calcium channel blockers are also probably effective at delaying preterm birth by 7 days (RR 1.15, 95% CI 1.04 – 1.27), however are probably more likely to result in cessation of treatment (RR 1.23, 95% CI 1.23 – 7.11). Importantly, calcium channel blockers possibly reduce the risk of adverse neonatal outcomes including neurodevelopmental morbidity (RR 0.51, 95% CI 0.30 – 0.85), respiratory morbidity (RR 0.68, 95% CI 0.53 – 0.88) and low birthweight (RR 0.49, 95% CI 0.28 – 0.87) [5]. As mentioned above, nitric oxide donors (13 trials) probably delay preterm birth and are ranked highest for delaying birth by 48 h and 7 days [5].

Human chorionic gonadotropin and relcovaptan were ranked medium potential (Fig. 4b). A placebo-controlled trial of 100 pregnant women suggested human chorionic gonadotropin can significantly delay labour [44] while two other trials (165 pregnant women) suggest it is as effective as magnesium sulfate at delaying labour for 48 h, with fewer side-effects [45, 46]. A pilot trial (18 women) of relcovaptan, an orally active vasopressin V1a receptor inhibitor, found it effectively reduced uterine contractions during premature labour compared to placebo [47], however development was discontinued in 2001 before the results of Phase II trials in Sweden, France and Poland were reported.

#### Phase I candidates

*Bryophyllum pinnatum* extract, a herbal succulent that contained phenolic constituents was ranked medium potential (Fig. 4c). The authors of a trial of 26 pregnant women comparing *Bryophyllum pinnatum* extract to nifedipine suggested it might be promising, but the study was stopped early due to poor recruitment [48].

#### Preclinical candidates

**Fig. 5:**
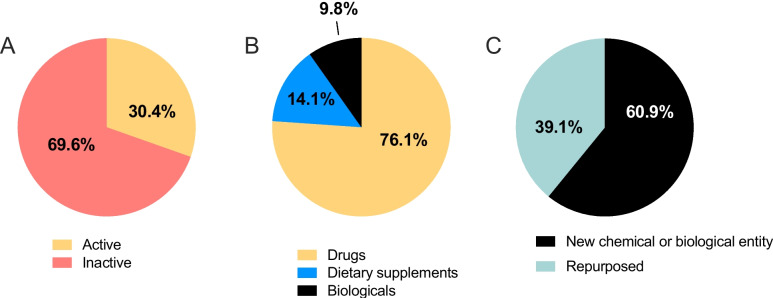
Details of the preclinical candidates in the R&D pipeline for preterm birth/labor. Summary of the preclinical candidates in the R&D pipeline for the prevention of preterm birth and treatment of preterm labor from 2000 – 2021. The proportion of candidates **A** in active development, and inactive (no publications since 2018), **B** classified as drugs, biologicals or dietary supplements, and **C** classified as new chemical or biological entities or repurposed drugs

Of the 107 candidates in preclinical development, 15 were excluded due to adverse effects, being inferior to other products in development or being used preclinically as a research tool to investigate pathophysiology and not intended for clinical translation. Of the 92 remaining candidates, 28 (30.4%) were active and 64 (69.6%) were inactive (Fig. 5A). Most candidates were drugs (70 candidates, 76.1%); 13 were dietary supplements (14.1%) and 9 were biologics (9.8%; Fig. 5B). There were 36 repurposed medicines (39.1%) and 56 new chemical or biological entities (60.9%; Fig. 5C). Overall, 34 candidates were evaluated for the prevention of preterm birth, 56 were evaluated for the management of preterm labour and two were evaluated for both the prevention and management (resveratrol and rolipram).

A diverse range of medicine subclasses were under investigation at the preclinical stage (Tables 2 and 3). The most common preclinical medicine subclass for prevention were amino acid/peptides (14 candidates, 38.9%; Table 2). Amino acid/peptides were also the most common subclass for management of preterm labour (12 candidates, 20.7%) followed by tocolytics and vascular agents (10 candidates each, 17.2%; Table 3). We identified 13 candidates with some concerns and six candidates with major concerns about the quality of the preclinical evidence. Concerns identified included lack of preterm birth animal model studies, conflicting results between studies of the same candidate and extremely high dose of candidate medicine used in preclinical studies.

**Table 2:** Summary of preclinical candidates for preterm birth prevention

**Drug subclass**	**Candidate**	**Summary**	**Archetype**	**Proposed administration**
*Prevention of preterm birth*				
Amino acid-peptide	AG126	A cell-permeable tyrophostin/tyrosine kinase inhibitor	New	Unspecified
	AG1288	A tyrosine kinase inhibitor	New	Unspecified
	Anti- Toll-like receptor 4 (TLR4) monoclonal antibody	Competitively binds to the TLR4 receptor, blocking TLR4-induced inflammation	New	Intravenous
	Etanercept	Dimeric fusion protein that binds specifically to TNF	Repurposed	Unspecified
	Histone deacetylase inhibitors (nanosuspension)	Anti-inflammatory histone deacetylase inhibitors delivered in a nanosuspension	New	Vaginal
	IMD-0560	Novel IκB kinase β inhibitor	New	Vaginal
	Melatonin	Hormone associated with the sleep–wake cycle	Repurposed	Oral
	NS-398	A selective COX-2 inhibitor with great specificity for prostaglandin-endoperoxide synthase 2	New	Intravenous
	Rytvela	Small, non-competitive IL-1R-biased ligand	New	Unspecified
	SB 202190	A highly selective, potent, cell-permeable p38 MAP kinase inhibitor	New	Unspecified
	SB 239063	A second-generation p38 MAP kinase inhibitor	New	Unspecified
	Super-repressor (SR) IχBα (exosome delivery)	NF-κB inhibitor delivered by engineered extracellular vesicles	New	Injection
	Synthetic TLR4	Synthetic TLR4 protein	New	Intravenous
	Vaginal progesterone (nanosuspension)	Natural progesterone delivered in a nanosuspension	New	Vaginal, rectal, topical
Antibiotics	Sirolimus	Macrolide antibiotic with potent anti-inflammatory effects	Repurposed	Oral
Anti-depressant	Rolipram	A selective phosphodiesterase-4 inhibitor	New	Oral, injectable
Cell therapy	Exosome-based protein therapeutics	Proprietary Exosome engineering for Protein loading via Optically reversible Protein–Protein interaction (EXPLOR™) technology	New	Injectable
	Pen-NBD (cell-penetrating peptide delivery)	Cell penetrating peptides (CPPs) are novel vectors that can traverse cell membranes without the need for recognition by cell surface receptors	New	Intravenous
Disease-modifying anti-rheumatic drugs	Sulfasalazine	Releases its breakdown product, 5-aminosalcyclic acid into the colon	Repurposed	Oral
Enzyme inhibitors (statins)	Simvastatin	Antilipemic agent from the statin family of drugs	Repurposed	Oral
Herbal	Abeliophyllum distichum Nakai leaf extract	A deciduous shrub native to South Korea being investigated for anticancer, antidiabetic, antihypertensive and anti-inflammatory activities	New	Oral
	Astragali radix extract	Dried root of Astragalus membranaceus Bunge. A traditional Chinese medicine	Repurposed	Oral
	Cucurbita moschata extract	Pumpkin extract	Repurposed	Oral
	Parthenolide	A sesquiterpene lactone occurring naturally in the plant feverfew (Tanacetum parthenium)	New	Oral; intravenous
Immunosuppressant	Tocilizumab	Treatment for rheumatoid arthritis	Repurposed	Intravenous, subcutaneous
Opioid receptor antagonist	( +)-Naloxone	Isomer of (-)-naloxone (Narcan), a medication indicated in opioid overdose	New	Intravenous, intramuscular, intraperitoneal, oral
	( +)-Naltrexone	Isomer of (-)-naltrexone (ReVia or Vivitrol), a medication used to manage opioid use	New	Intravenous, intramuscular, intraperitoneal, oral
Organic compound	U-0126	Inhibits activation of MAPK (ERK1/2) by inhibiting kinase activity of MAP Kinase Kinase	New	Subcutaneous
Polyphenol	Gallic acid	A trihydroxy benzoic acid found in gallnuts, sumac, witch hazel, tea leaves, oak bark and other plants	Repurposed	Oral
	Honokial	A polyphenol lignan. The primary component of the traditional Chinese medicinal herb Houpo	Repurposed	Oral
	Nobiletin	A polymethoxy flavone abundant in citrus flavedo	Repurposed	Oral
	Resveratrol	A stilbenoid produced by several plants in response to injury	Repurposed	Oral
Probiotics	Microbiome therapeutics	Live biotherapeutics being developed for preterm labour	New	Unspecified
Small molecule	Sc514	Small molecule which targets the intracellular NF-κB pathway	New	Unspecified
	TPCA-1	IKKβ inhibitor	New	Unspecified
Unclassified	Replens gel	A bioadhesive vaginal moisturiser, and the vehicle for vaginal progesterone	Repurposed	Vaginal

**Table 3:** Summary of preclinical candidates for preterm labour management

**Drug subclass**	**Candidate**	**Summary**	**Archetype**	**Administration**
*Management of preterm labor*				
Amino acid-peptide	15d-PGJ2	An anti-inflammatory prostaglandin	New	Intravenous
	Azapeptide analogues	Azapeptides are peptide analogs in which one or more of the amino residues is replaced by a semicarbazide	New	Subcutaneous
	BRL 37344	A selective agonist of the β3 adrenergic receptor	New	Unspecified
	Butaprost	A structural analogue of PGE2	New	Unspecified
	CyPPA	Stimulates myometrial Ka(Ca)2.2/2.3 channels to supress Ca^2+^-mediated uterine contractions	New	Injectable
	Exedine-4	A 39 amino-acid polypeptide isolated from Gila monster lizard saliva	Repurposed	Subcutaneous, intravenous
	Leptin	Hormone synthesised in adipose tissue, involved in regulation of energy balance, metabolism and body weight	Repurposed	Oral, subcutaneous
	N-acetylcysteine (nanoparticle delivery)	A precursor to glutathione delivered via targeted dendrimer nanoparticles	New	Intravenous
	PDC113.824	An experimental peptidomimetic prostoglandin F2α receptor ligand	New	Injectable
	SCH-772984	A selective inhibitor of ERK1/2	New	Unspecified
	SKF-86002	A p38 MAPK inhibitor	New	Unspecified
	Surfactant protein A	Protein component of surfactant, produced in the fetal lung	New	Injection
Anti-depressant	Rolipram	A selective phosphodiesterase-4 inhibitor	New	Oral, injectable
Anti-convulsant	Retigabine	An adjuvant therapy to treat partial epilepsies	Repurposed	Oral
Anti-malarial	Chloroquine/hydroxychloroquine	An aminoquinolone derivative developed to treat malaria	Repurposed	Intravenous, oral
Herbal	Carvacrol	A phenolic monoterpenoid found in essential oils of oregano, thyme, pepperwort, wild bergamot and other plants	New	Unspecified
	Ananas comosus, ethyl acetate fraction	Pineapple extract and traditional medicine	Repurposed	Oral
	Curcuma aeruginosa rhizome	A common medicinal plant used in Southeast Asia	Repurposed	Unspecified
	*Pimpinella anisum* extract	Anise, a common traditional medicine	Repurposed	Oral
	Paeoniflorin	A component of the dried root extract of Paeonia lactiflora Pall used widely in China, Korea, and Japan's traditional medicine	New	Intravenous
Hydrogen sulfide donors	GYY4137	Sulphide-releasing aspirin	New	Intravenous
Muscle relaxant	Botulinum toxin A	A neurotoxic protein, commonly known as Botox	Repurposed	Intramuscular, intravenous
Organic compound	1,10-Phenatroline	Heterocyclic organic compound that targets bitter taste receptors	New	Unspecified
	Alpha-bisabolol	An unsaturated, optically active sesquiterpene alcohol distilled from plant essential oils	Repurposed	Unspecified
	Citral	A pair, or mixture of terpenoids present in the oils of several plants including lemons, organges, limes and lemongrass	Repurposed	Oral, intravenous
	OXznl	A resorcylic acid lactone derived from fungus	New	Injectable
Polyphenol	Galetin 3,6-dimethyl ether	A Brazilian folk medicine	Repurposed	Intravenous, oral
	Resveratrol	A stilbenoid produced by several plants in response to injury	New	Unspecified
	Scutellaria baicalensis root extract	Tocolytic traditional Chinese medicinal herb	Repurposed	Oral
	Tannic acid	A weak acid found in nutgalls formed by insects on twigs of certain oak trees	Repurposed	Oral, vaginal, topical
Proton-pump inhibitors	Esomeprazole	Medication to reduce stomach acid, treatment for gastro-oesophageal reflux disease (GERD)	Repurposed	Oral, intravenous
	Lansoprazole	Medication to reduce stomach acid	Repurposed	Oral, intravenous
	Omeprazole	Medication to reduce stomach acid	Repurposed	Oral, intravenous
	Pantoprazole	Medication to reduce stomach acid	Repurposed	Oral, intravenous
	Rabeprazole	Medication to reduce stomach acid	Repurposed	Oral, intravenous
Thalidomide analogue	4APDPMe	Phosphodiesterase-4 inhibitor	New	Intravenous, intramuscular
	4NO2DPDMe	Phosphodiesterase-4 inhibitor	New	Intravenous, intramuscular
Tocolytic	AS603831	Non-peptide oxytocin receptor antagonist	New	Oral, intravenous
	AS604872	Prostaglandin F2α receptor antagonist	New	Oral
	HC067047	Transient receptor potential vanilloid 4 inhibitor	New	Intraperitoneal
	Hydrozone sulfanilide oxytocin antagonists	Class of oxytocin antagonists with high degree of selectivity against the closely related vasopressin receptors	New	Intravenous
	Indomethacin (nanoparticle delivery)	Nanoparticle preparation of a highly potent tocolytic	New	Oral
	PGN-1473	Prostaglandin EP2 receptor agonist	New	Intrauterine
	PGN-9856	Prostaglandin EP2 receptor agonist	New	Injectable
	Salbutamol (nanoparticle delivery)	Bronchodilator used to treat asthma and COPD used off label as a tocolytic, nanoparticles delivered via liposome	Repurposed	Unspecified
	SAR-150640	Selective β3 adrenergic receptor	New	Parenteral
	THG113.31	Selective prostaglandin F2-α antagonsist	New	Intravenous, topical
Uricosuric agent	Benzbromarone	Non-competitive inhibitor of xanthine oxidase used in the treatment of gout	Repurposed	Oral
Vascular agents	Amiloride	An antihypertensive	Repurposed	Oral, intraperitoneal
	Isradipine	Calcium-channel blocker related to nifedipine	Repurposed	Oral
	LDD175	BK(Ca) channel opener	New	Injectable
	Levosimendan	A hydrozone and pyridazine derivative	Repurposed	Intravenous, oral
	MONNA	Aanoctamin 1 antagonist	New	Unspecified
	Nebivolol	A third generation, FDA‐approved β1‐adregneric receptor (β1AR) antagonist	Repurposed	Oral
	Nifedipine (nanoparticle delivery)	PEGylated liposomes loaded with the potent tocolytic nifedipine	New	Intravenous, oral, rectal
	Pinacidil	A cyanoguanidine drug that acts by opening ATP-sensitive potassium channels	Repurposed	Oral
	S-Nitrocysteine	A low molecular weight, cell-permeable nitrosothiol and nitric oxide donor	New	Injectable
	ZD-7288	A specific bradycardic agent	New	Unspecified

## Discussion

### Main findings

We systematically analysed the medicines R&D pipeline for preterm birth/labor between 2000 and 2021. Of the 178 candidates approximately 4% have made it to market for this indication. Novel medicines accounted for 46% of candidates in clinical development for management of preterm labour, a substantially higher proportion compared to other maternal conditions [25]. However, we identified only one novel medicine – oral 17-alpha-hydroxyprogesterone caproate—in clinical development for preterm birth prevention. Through matching candidates to pre-specified TPP criteria, we identified six high priority candidates for spontaneous preterm birth prevention (omega-3 fatty acids, aspirin, vaginal and oral progesterone, pravastatin, l-arginine and selenium) and four high priority candidates for management of preterm labour (nicorandil, isosorbide dinitrate, nicardipine and celecoxib), which warrant R&D investment.

### Interpretation in light of other evidence

A 2008 analysis of the obstetric R&D pipeline identified 67 candidates in development for maternal conditions between 1980 and 2007 [13]; in contrast, we identified 444 candidates in development for five obstetric conditions between 2000 and 2021. Both studies found preterm birth/labor to be the dominant indication (45% and 40%, respectively), contributing the greatest number of individual candidates in the pipeline [13]. Given that preterm birth is the leading cause of mortality in newborns and children globally, this is perhaps unsurprising [49]. In high-income countries, where the majority of global research funding is spent [50], preterm birth is a leading cause of long-term disability and imposes significant societal and financial costs [51].

Most candidates in the preterm birth pipeline that had made it to market or were used extensively off-label were tocolytics. Our analysis identified four high- and five medium-potential candidate tocolytics, in addition to the tocolytics currently available. Strikingly, all high potential and two medium potential candidates are from the same drug classes as known effective tocolytics, including COX-inhibitors (celecoxib), nitric oxide donors (isosorbide dinitrate, glyceryl trinitate), oxytocin receptor antagonists (retosiban) and calcium channel blockers (nicorandil, nicardipine) [6]. Our analysis highlights the relatively strong interest in R&D for improved tocolytics, likely because of the efficacy evidence on drugs within the same class and that the mechanisms underlying labour progression are well characterised, providing specific drug targets [52].

In contrast, there are currently few medicines that are recommended for the prevention of preterm birth. A significant amount of active R&D concerns progestin medicines for preterm birth prevention. Studies have investigated different formulations and routes of administration (such as vaginal and oral natural micronized progesterone), as well as aiming to identify which sub-population of women will benefit most from progesterone [8]. Recently, evidence was in favour of using vaginally administered progesterone in women at increased risk of preterm birth, particularly women with a short cervix [8], and hence some guidelines recommended its use for preventing preterm birth in women with a history of preterm birth and/or cervical shortening [53–55]. However, a 2022 meta-analysis found that progesterone was not effective in preventing recurrent preterm birth in women with a history of spontaneous preterm birth, in the absence of cervical shortening [56]. Assessment of cervical length requires access to transvaginal ultrasound, which is not available in all settings. Two meta-analyses from 2021 found no significant effect of injectable 17-alpha-hydroxyprogesterone caproate in preventing preterm birth, prompting the FDA, in 2022, to recommend its withdrawal from market [8, 57]. These recent additions to the body of evidence on progesterone’s efficacy have prompted updated guidance from American College of Obstetricians and Gynecologists (ACOG) on use of progesterone in pregnant women [58]. Pending the FDA final determination, hydroxyprogesterone caproate injection continues to be recommended by the International Federation of Obstetrics and Gynaecology (FIGO) in women at risk of spontaneous preterm birth [53].

Dietary supplements represented 18% of the candidates under investigation for preterm birth. Trials of some of these supplements, including vitamins A, C and E, have been conclusively shown not to be effective at preventing preterm birth [59–61]. In contrast, omega-3 fatty acids, L-arginine and selenium supplementation all have promising clinical efficacy evidence, though further evidence is needed [27, 36, 38]. There are clear implementation advantages for dietary supplements – they are usual taken orally, many are low cost and widely available, and they may be viewed as natural supplements rather than drugs, which could be more acceptable to women [62]. However, questions remain about the population of women who would benefit most from these supplements, and whether companion diagnostic tests for vitamin and mineral deficiencies are required [63] which can be a barrier to implementation.

The large number of candidates in development for preterm birth/labor suggests comparatively high R&D interest for this condition. However, 62% of all candidates and 72% of candidates in preclinical development for preterm birth/labor are inactive, with no updates or published progress since 2018. For example, the promising oxytocin receptor antagonist retosiban developed by GSK was halted in Phase III trials due to difficulties with trial recruitment [43]. The complexities of conducting large, well-designed clinical trials in pregnant women is a recognised barrier inhibiting R&D for maternal conditions [15]. A 2013 review of all US-based, phase IV, pharmaceutical industry-sponsored clinical trials that included women of childbearing age between 2011–2012, found that only 1% were specifically designed for pregnant women, and 95% specifically excluded pregnant women [64]. Pharmaceutical industry representatives cite potential liability issues, additional risks related to teratogenicity and the prohibitively large sample sizes needed to demonstrate benefit as the reasons for the lack of R&D in maternal medicines [15]. To overcome these barriers, broader international coordinated efforts around high potential candidates is needed. Successful strategies have been implemented to overcome similar barriers in the development of medicines for children. For example, in 2007, “Paediatric Investigation Plans” (PIP) were introduced by the European Union, obliging companies applying for licences for new medicines to present a plan to study the medicine in children (unless inappropriate for this age group). This scheme greatly improved the product pipeline for children’s medicines, leading to over 260 new medicines or indications for children since its launch [65]. There remains an urgent need for large trials for candidate medicines for the prevention of preterm birth and the management of spontaneous preterm labor, which are adequately powered for clinically relevant neonatal outcomes.

### Strengths and limitations

We have developed a novel, drug-agnostic approach for analysing the R&D pipeline for medicines for spontaneous preterm birth and preterm labor. Our analysis provides the most extensive mapping of the historic and current R&D pipeline for preterm birth/labor and identified ten high priority candidates currently in clinical development, as well as significant gaps in R&D. This approach may be useful for prioritising research for other maternal conditions, as well as other fields of drug development, particularly where TPPs already exist [18]. However, there are some limitations to this approach. Firstly, ranking of candidates relied on available information, and it is possible that data on some candidates are not publicly available. Secondly, this system of matching candidates to the TPPs was not possible for candidates in preclinical development, due to a lack of available data on many variables in the TPPs. Thus, when examining preclinical candidates, other factors such as the quality of the laboratory findings should be considered prior to further investments in clinical trials. Finally, preterm birth is a broad category that can involve many different aetiologies. It is highly unlikely that a single drug could be effective in all forms of preterm birth. All medicines under investigation for preterm birth were included in the database, as the specific aetiology an individual medicine was targeting was often poorly articulated. In the current study we analysed each candidate medicine against the TPPs for medicines to prevent spontaneous preterm birth, as this was highlighted during TPP development as the specific target population most in need [16]. As advances are made in the understanding of the causes of preterm birth the TPP, a living document, and the pipeline analysis can be updated to address additional pathologies.

## Conclusion

Over the last 20 years R&D for preterm birth/labor medicines is an active area compared to other maternal conditions. However, many candidates, including promising new therapies, are currently inactive. Development of alternative tocolytics, new formulations of progestins and dietary supplements are areas of high research activity. We identified six high priority candidates for preventing spontaneous preterm birth and four high priority candidates for the management of preterm labor that best meet real-world requirements. This novel method of matching drug candidates to TPPs can help better direct research funding towards the most promising candidates and ensure new and effective therapies become available.

## Supplementary Information

**Additional file 1: Appendix A.** Supplementary tables. **Table S1.** Scoring of target product profile comparison, for quantification of potential of candidates. **Table S2.** Threshold for ranking of potential at each phase of the R&D development pipeline.

**Additional file 2: Appendix B.** Low rank candidates.

## Data Availability

All data relevant to the study are included in the article or uploaded as supplementary information. The database generated and analysed during the current study are available at https://www.conceptfoundation.org/accelerating-innovation-for-mothers/.

## Abbreviations

ACOG: American College of Obstetricians and Gynecologists
AIM: Accelerating Innovation for Mothers
COX: Cyclooxygenase
FDA: Food and Drug Administration
FIGO: Fédération Internationale de Gynécologie et d'Obstétrique
GSK: GlaxoSmithKline plc.
R&D: Research and development
TPP: Target product profile
WHO: World Health Organization
