# Supplementary material for: New medicines for spontaneous preterm birth prevention and preterm labour management: landscape analysis of the medicine development pipeline
Source: BMC Pregnancy Childbirth. 2023 Jul 18;23:525. doi: 10.1186/s12884-023-05842-9 (PMC10354994; doi:10.1186/s12884-023-05842-9)
Supplement: Supplementary file 1 — Additional file 1: Appendix A. Supplementary tables. Table S1. Scoring of target product profile comparison, for quantification of potential of candidates. Table S2. Threshold for ranking of potential at each phase of the R&D development pipeline. [file 12884_2023_5842_MOESM1_ESM.docx]

Appendix A: supplementary tables

Table S1: Scoring of target product profile comparison, for quantification of potential of candidates.

| **Variable** | **Answer options** | **Coding value** |
| --- | --- | --- |
| Target country | Not stated | 0 |
|  | HIC only | 1 |
|  | LMIC only | 1 |
|  | HIC and LMIC | 2 |
| Efficacy | Not yet known | 0 |
|  | Not met minimum | AUTOMATIC LOW |
|  | Partially met minimum | 1 |
|  | Met minimum | 3 |
|  | Met preferred | 5 |
| Safety | Not yet known | 0 |
|  | Not met minimum | AUTOMATIC LOW |
|  | Partially met minimum | 1 |
|  | Met minimum | 2 |
|  | Met preferred | 3 |
| Companion Diagnostic, | Not yet known | 0 |
| Monitoring, | Not met minimum | -2 |
| Adherence | Partially met minimum | 1 |
| and Administration | Met minimum | 2 |
|  | Met preferred | 3 |
| Stability (is cold chain storage required?) | Yes | -2 |
|  | No | 2 |
|  | Unsure | 0 |
| WHO EML (is candidate currently on the EML list?) | Yes | 1 |
|  | No | -1 |
|  | Unsure | 0 |

Table S2: Threshold for ranking of potential at each phase of the R&D development pipeline

| **Final ranking** | **Phase III** | **Phase II** | **Phase I** |
| --- | --- | --- | --- |
| *High potential* | >17 | >13 | >13 |
| *Medium potential* | 9-16 | 9-13 | 6-13 |
| *Low potential* | <9 | <9 | <6 |
