# Supplementary material for: New medicines for spontaneous preterm birth prevention and preterm labour management: landscape analysis of the medicine development pipeline
Source: BMC Pregnancy Childbirth. 2023 Jul 18;23:525. doi: 10.1186/s12884-023-05842-9 (PMC10354994; doi:10.1186/s12884-023-05842-9)
Supplement: Supplementary file 2 — Additional file 2: Appendix B. Low rank candidates. [file 12884_2023_5842_MOESM2_ESM.docx]

Appendix B: Low rank candidates

PREVENTION OF PRETERM BIRTH

Phase III - Probiotic lactobacilli, vitamin D, pentaerythrityl tetranitrate, and Zishen Yutai pill all ranked low potential. Probiotic lactobacilli have been proposed to reduce the risk of preterm birth by maintaining a healthy vaginal microbiome, however the clinical efficacy remains unknown. As of 2022, there are four clinical trials registered as “recruiting”, investigating the effects of oral or vaginal probiotic lactobacilli on preterm birth. A 2019 Cochrane review demonstrate that Vitamin D supplementation does not meet the minimum requirements for clinical efficacy.^1^ There is limited clinical evidence for the effectiveness and safety pentaerythrityl tetranitrate and Zishen Yutai pills during pregnancy.

Phase II - The remaining candidates ranked low potential. Oral N-acetylcysteine, an amino acid, in combination with progesterone has been found to significantly prolong gestation in pregnant women treated for bacterial vaginosis, compared to progesterone alone.^2^ However N-acetylcysteine failed to meet the minimum requirement for companion diagnostics. Although zinc, lycopene and iodine supplementation all met the preferred or minimum requirements for the majority of variables, they failed to meet the minimum requirement for clinical efficacy. Meta-analysis of 16 trials between 1977 and 2008 concluded there was low quality evidence for a modest reduction in risk of preterm birth with maternal zinc supplementation (sRR 0.86, 95% CI 0.75 – 0.99; 7818 births), but that zinc has no effect on gestational age.^3^ Meta-analysis of iodine supplementation in pregnant women found low quality evidence of no effect of iodine on the risk of preterm birth (RR 0.71, 95% CI 0.30 – 1.66, 2 trials, 376 women).^4^ Alpha-lipoic acid, vitamin B12 and oral 17-alpha-hydroxyprogesterone caproate all ranked low due to limited evidence of their clinical efficacy and matching to other variables. A phase II trial of oral 17-alpha-hydroxyprogesterone caproate is registered in the USA (ref). It is noteworthy that while the efficacy and safety of oral 17-alpha-hydroxyprogesterone caproate is not yet known, it is being developed to overcome the administration difficulties of injectable 17-alpha-hydroxyprogesterone caproate, which is on the market and has been shown to prevent preterm birth^5^ (though there has subsequently been a lack of benefit demonstrated in post-market trials).

Phase I - Aminophylline ranked low potential due to a lack of clinical evidence, and ulinastatin (urinary trypsin inhibitor) ranked low potential due a small trial of 92 women with short cervical length, which found ulinastatin did not have any effects on preterm birth compared to placebo.^6^

MANAGEMENT OF PRETERM LABOUR

Phase III - Dydrogesterone was ranked low potential, due to a failure to meet the minimum requirements for clinical efficacy. Two randomized trials reported that adjunctive treatment oral dydrogesterone did not prolong latency period in preterm labour when compared to placebo.^7,8^ Meluadrine tartrate, a selective β2-adrenoceptor agonist was also ranked low. Phase III trials of Meluadrine tartrate were conducted in 1998 by Tokyo Tanabe, however trial data is not publicly available, and development has been discontinued.

Phase II - Sulidac and barusiban both ranked low due to failure to meet the minimum requirements for clinical efficacy. Sulidac, a non-selective non-steroidal anti-inflammatory drug has been trialled as an oral therapy to prevent premature labour reoccurrence after successful tocolysis. Evidence from three trials (208 women) all found no difference in readmission for tocolysis in women taking sulindac compared to placebo.^9-11^ Barusiban, an oxytocin receptor agonist similar to atosiban, was found to be not effective at reducing uterine contractions or delaying preterm birth (one trial, 163 women).^12^ L-arginine ranked low as the clinical efficacy is unknown. A Polish trial in 70 women with threatened preterm delivery found no difference in gestational age at birth between L-arginine + tocolytic therapy and tocolytic therapy alone.^13^ The remaining candidates (ebopiprant, montelukast, nolasiban, ketoprofen and bedoradine) ranked low due to a lack of available data on most TPP variables.

Phase I - Sildenafil citrate, ONO 8815Ly, TT 235 and SSR-126768A all ranked low due to a lack of available data on most TPP variables.

REFERENCES

1. Palacios C, Kostiuk LK, Pena-Rosas JP. Vitamin D supplementation for women during pregnancy. *Cochrane Database Syst Rev* 2019; **7**: CD008873.

2. Shahin AY, Hassanin IM, Ismail AM, Kruessel JS, Hirchenhain J. Effect of oral N-acetyl cysteine on recurrent preterm labor following treatment for bacterial vaginosis. *Int J Gynaecol Obstet* 2009; **104**(1): 44-8.

3. Chaffee BW, King JC. Effect of zinc supplementation on pregnancy and infant outcomes: a systematic review. *Paediatric and perinatal epidemiology* 2012; **26 Suppl 1**: 118-37.

4. Harding KB, Pena-Rosas JP, Webster AC, et al. Iodine supplementation for women during the preconception, pregnancy and postpartum period. *Cochrane Database Syst Rev* 2017; **3**: CD011761.

5. Group E. Evaluating Progestogens for Preventing Preterm birth International Collaborative (EPPPIC): meta-analysis of individual participant data from randomised controlled trials. *Lancet* 2021; **397**(10280): 1183-94.

6. Otsuki K, Kawabata I, Matsuda Y, et al. Randomized trial of the efficacy of intravaginal ulinastatin administration for the prevention of preterm birth in women with a singleton pregnancy and both cervical shortening and inflammation of lower genital tract. *J Obstet Gynaecol Res* 2019; **45**(1): 86-95.

7. Areeruk W, Phupong V. A randomized, double blinded, placebo controlled trial of oral dydrogesterone supplementation in the management of preterm labor. *Sci Rep* 2016; **6**: 20638.

8. Thongchan S, Phupong V. Oral dydrogesterone as an adjunctive therapy in the management of preterm labor: a randomized, double blinded, placebo-controlled trial. *BMC pregnancy and childbirth* 2021; **21**(1): 90.

9. Bartfield M, Carlan SJ. The safety and efficacy of prolonged outpatient sulindac to prevent the recurrence of preterm labor: a prospective double-blind study. *Prim Care Update Ob Gyns* 1998; **5**(4): 178.

10. Carlan SJ, O'Brien WF, Jones MH, O'Leary TD, Roth L. Outpatient oral sulindac to prevent recurrence of preterm labor. *Obstet Gynecol* 1995; **85**(5 Pt 1): 769-74.

11. Humphrey RG, Bartfield MC, Carlan SJ, O'Brien WF, O'Leary TD, Triana T. Sulindac to prevent recurrent preterm labor: a randomized controlled trial. *Obstet Gynecol* 2001; **98**(4): 555-62.

12. Thornton S, Goodwin TM, Greisen G, Hedegaard M, Arce JC. The effect of barusiban, a selective oxytocin antagonist, in threatened preterm labor at late gestational age: a randomized, double-blind, placebo-controlled trial. *Am J Obstet Gynecol* 2009; **200**(6): 627 e1-10.

13. Rytlewski K, Olszanecki R, Lauterbach R, et al. Effects of oral L-arginine on the pulsatility indices of umbilical artery and middle cerebral artery in preterm labor. *Eur J Obstet Gynecol Reprod Biol* 2008; **138**(1): 23-8.
